# Facile Synthesis of Solution-Processed Silica and Polyvinyl Phenol Hybrid Dielectric for Flexible Organic Transistors

**DOI:** 10.3390/nano10040806

**Published:** 2020-04-23

**Authors:** Xiong Chen, Yu Zhang, Xiangfeng Guan, Hao Zhang

**Affiliations:** 1 Organic optoelectronics research center in Fujian Universities, College of electronics and information science, Fujian Jiangxia University, Fuzhou 350108, China; chenxiong@fjjxu.edu.cn (X.C.); zhangyu@fjjxu.edu.cn (Y.Z.); xfguan@fjjxu.edu.cn (X.G.); 2 College of Materials Science and Engineering, Shanghai University, Shanghai 200444, China

**Keywords:** sol–gel method, silica, high dielectric constant, organic thin film transistors

## Abstract

A high-quality dielectric layer is essential for organic thin-film transistors (OTFTs) operated at a low-power consumption level. In this study, a facile improved technique for the synthesis of solution-processed silica is proposed. By optimizing the synthesis and processing technique fewer pores were found on the surface of the film, particularly no large holes were observable after improving the annealing process, and the improved solution–gelation (sol–gel) SiO*_x_* dielectric achieved a higher breakdown strength (1.6 MV/cm) and lower leakage current density (10^−8^ A/cm^2^ at 1.5 MV/cm). Consequently, a pentacene based OTFT with a high field effect mobility (~1.8 cm^2^/Vs), a low threshold voltage (−1.7 V), a steeper subthreshold slope (~0.4 V/dec) and a relatively high on/off ratio (~10^5^) was fabricated by applying a hybrid gate insulator which consisted of improved sol–gel SiO*_x_* and polyvinyl phenol (PVP). This could be ascribed to both the high k of SiO*_x_* and the smoother, hydrophobic dielectric surface with low trap density, which was proved by atomic force microscopy (AFM) and a water contact angle test, respectively. Additionally, we systematically studied and evaluated the stability of devices in the compressed state. The devices based on dielectric fabricated by conventional sol–gel processes were more susceptible to the curvature. While the improved device presented an excellent mechanic strength, it could still function at the higher bending compression without a significant degradation in performance. Thus, this solution-process technology provides an effective approach to fabricate high-quality dielectric and offers great potential for low-cost, fast and portable organic electronic applications.

## 1. Introduction

In recent years, the search for a technology diverging from the conventional rigid silicon technology has intensely stimulated fundamental scientific and technological research efforts [1,2,3,4]. These efforts may take organic thin film transistors (OTFTs) fabricated on the unbreakable substrates as viable alternatives to amorphous Si-based devices. How to improve the device performance of OTFTs is still a challenge for speeding up its practical application. In particular, as a key parameter threshold voltage (*V_th_*), which is especially important for portable electronics, needs to be further decreased. As we know, the surface properties and the dielectric constant (*k*) of gate insulators play a dominant role in threshold voltage. The surface properties have a strong influence on the trap–state density related to the threshold voltage [5,6]. The k value of a gate insulator directly determines the capacitance value, which leads to the change of threshold voltage [7]. As a kind of classic dielectric material, silicon with thermal oxide (SiO_2_) has been widely used as the gate insulator in OTFTs. It also provides a high-quality substrate (smooth surface and relatively good thermal and chemical stability) to grow organic, semiconducting thin-film. However, the SiO_2_ thin-film is usually prepared on the Si wafer by thermal oxidation techniques which possess some limitations in mass production and in realization in large-area electronics, in particular it is not suitable for those flexible devices processed at low temperatures. Moreover, the low k of SiO_2_ (~3.0) also brings a high threshold voltage that is unfavorable for practical applications.

In this case, a facile solution-process that has the two advantages of low-temperature and a large-area process is attractive. Solution-processed metal oxide semiconductors have made enormous progress in the past few years [8,9,10]. However, the film prepared by the solution method has the general problem of a large leakage current, especially for the silica film prepared by solution–gelation (sol–gel) [11,12]. Due to the holes or pores generated during film preparation, the switching current of related devices is relatively low at present. Meanwhile, there is no relevant research on whether the silica film prepared by solution processing is suitable for the bending requirements of emerging electronics.

In this work, we propose a facile solution processing technique for synthesis of silica by optimizing the synthesis and processing, fabricating a high-quality hybrid dielectric (improved sol-gel SiO*_x_*/PVP) for organic transistors. The resulting OTFT device exhibits a low threshold voltage (~−1.7 V) and subthreshold slope (~0.4 V/dec), a relatively high on/off ratio (~10^5^). Moreover, we systematically studied and tested the stability of devices fabricated by the improved solution method in the flexible state.

## 2. Materials and Methods

### 2.1. Materials Synthetic

Reagent graded chemicals of Tetraethylorthosilicate (TEOS), isopropyl alcohol, nitric acid and distilled water were used as starting materials to prepare the solution. At the beginning, 2.5 mL of TEOS was dissolved in 15 mL of isopropyl alcohol for 60 min. 1 mL of distilled water was then mixed up and added to 0.2 mL of acetic acid for catalysis. After stirring for 60 min, a clear solution was formed. The time and temperature for stirring and annealing have an important influence on the synthesis of materials, so these parameters must be precisely controlled and consistent in the synthesis of all samples. PVP (polyvinyl phenol, Mw~25,000) (20 wt%) and poly (melamine-co-formaldehyde) (MMF) (10 wt%) were dissolved in propylene glycol monomethyoether acetate (PGMEA). The MMF was used herein as the cross-linker of PVP to remove its hydroxyl groups. 

### 2.2. Device Fabrication

The conversion of the sol−gel to the substrates was adopted as follows: firstly, the prepared sol−gel solution was dropped onto the substrates and spin-coated at 3000 rpm for 30 s; next, the substrates were placed on a hot plate at 120 °C for 1 h and then the resulting film was cross-linked under UV light for 10 min in the ozone cleaner. To improve the quality of sol–gel film, we controlled the heating temperature and prolonged the film formation process (60 °C for 12 h) in which the contained water and solvent were slowly evaporated. The final curing was done for 1 h at 120 °C. For comparison, an ultra-thin film of PVP (~20 nm) was additionally spin-coated onto the improved sol–gel film as a hybrid dielectric layer.

**Figure 1:**
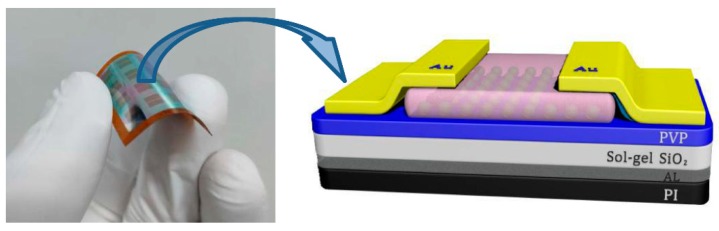
The schematic structures of fabricated organic thin film transistors (OTFTs) device based on polyimide (PI) substrate.

Bottom-gate, top-contact OTFTs were fabricated on a polyimide (PI) substrate for flexibility tests. The corresponding device configuration is shown in Figure 1. Initially, aluminum (~100 nm) serving as the gate electrode was deposited by thermal evaporation on the PI substrate, which was treated with ultraviolet (UV) ozone for 3 min. The sol–gel SiO*_x_* and PVP solution were deposited onto the substrate by spin-coating in a glove box filled with nitrogen. The thicknesses of SiO*_x_* and PVP were 150 and 20 nm as measured by profilometer, respectively. Two thermal treatment steps were used in the process of SiO*_x_* (120 °C for 1 h,) and PVP (120 °C for 1 h). Afterward, the n-type pentacene (Sigma Aldrich, Shanghai, China) (~40 nm) was then deposited at a rate of approximately 1 Å/s under a background pressure of 10^−4^ Pa. The wafers were then rapidly transferred to a vacuum chamber to define the source and drain contacts by thermally evaporating Au (~30 nm) through a shadow mask with a background pressure of 10^−4^ Pa. The width and length of the device channel were defined as 1500 and 50 μm, respectively.

### 2.3. Device Characterization

Crystal phase structures of the pentacene were characterized by an MiniFlex 600 X-ray diffraction (XRD) instrument (Rigagu, Tokyo, Japan) over the 2*θ* of 10° to 80°. The surface energies of insulators were established by measuring the contact angle using a kino SL200 KS goniometer (Kino, NY, USA). Electrical characteristics of OTFTs were performed by a Keithley 4200-SCS semiconductor parameter analyzer (Tektronix, Johnston, OH, USA) in ambient. The atomic force microscopy (AFM) images were obtained by an SPI 400 atomic force microscope (HITACHI, Tokyo, Japan) in tapping mode.

## 3. Results and Discussion

**Figure 2:**
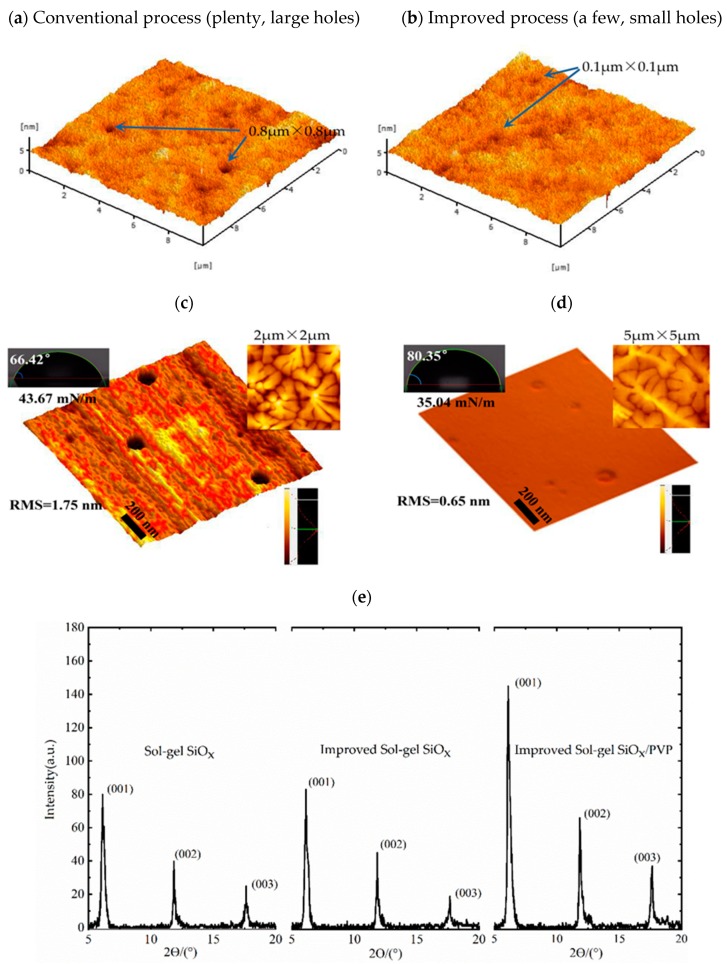
Three-dimensional topography images of (**a**) conventional solution–gelation (sol–gel) SiO*_x_*, (**b**) improved sol–gel SiO*_x_*, (**c**) upscaled improved sol–gel SiO*_x_* film, (**d**) upscaled improved sol–gel SiO*_x_*/PVP film. Water contact angle (left), atomic force microscope (AFM) image of pentacene grown on related dielectric(right). (**e**) X-ray diffraction (XRD) patterns of crystal phase structures of pentacene (40 nm) grown on different dielectrics.

A topographical characterization of the dielectric films is depicted in Figure 2. By the conventional sol–gel process, there were plenty of holes with a diameter of more than ~0.2 μm and a typical depth of 30 nm, and we even found two of these holes with an area of ~0.8 μm × 0.8 μm (Figure 2a). During processing, small particles are formed that are dispersed in the liquid. They agglomerate to form a three-dimensional network of Si−O−Si bonds and is designated as a gel, the structure of the gel is dependent on the time of gelation. When we controlled the heating temperature and prolonged the film formation process, fewer holes were found, particularly, no large holes were observable. We consider that these holes were generated by the evaporation of the solvent and by the remaining gas from chemical reactions during the annealing process. After improving the annealing process, the lower heating temperature slowed down the rate of evaporation of gas and solvent and also of the gelation process. We assume that the improved method extends the process of the final film curing, which offers enough time for its network to bind more tightly together after the gas discharge. Therefore, the pore shrinkage rate increases noticeably. 

To further reduce the influence of porosity on the dielectric layer, it was coated with an ultra-thin crosslinking PVP. Figure 2c,d shows that a remaining small amount of pores (less than a diameter of 100 nm) were covered by the viscous polymer solution, which filled up the valley regions of the sol–gel SiO*_x_* gate dielectric surface. Hence, fewer upheavals can be seen on the surface, which leads to the surface roughness reducing from 1.75 to 0.65 nm. The polymer offers a smoother surface for the growth of pentacene. As seen in the insets of Figure 2c,d, when grown on PVP-coated gate dielectric, pentacene exhibits well-formed terraces and large dendritic grains. Clearly, larger grains are obtained as the surface roughness is decreased by the polymer films. This can be attributed to the increased diffusion length of the pentacene molecules, as well as the increased energy barrier for nucleation during the formation of nuclei. This morphology provides low trap-state density by lowering the grain boundary and increasing grain size comparing with granular grains obtained from the bare sol–gel SiO*_x_* substrate. On the other hand, inorganic oxides with high polarizabilities possess a high density of surface –OH groups, presenting hydrophilic surfaces. These surfaces with a high polar surface energy tend to have low water angles [13]. The measured water contact angles are 66.42°, 80.35° for sol–gel SiO*_x_* and PVP modified sol–gel SiO*_x_* gate dielectric, respectively. Notably, after treatment with cross-linked PVP the water contact angle increases, the surface was transformed into a relatively hydrophobic surface and now exhibits a dramatically reduced abundance of –OH groups, as evidenced by the lower surface energy values measured for the sol–gel SiO*_x_*/PVP gate dielectric (35.04 mN/m). 

X-ray diffraction (XRD) was tested for the growth of pentacene on different dielectric layers (Figure 2e). A sharp diffraction peak appears at 2*θ* = 5.56°, corresponding to the (001) crystal plane of pentacene. The diffraction peak intensity of (001) direction of pentacene grown on the surface of conventional and improved sol–gel SiO*_x_* film is at the nearly same value. After being coated with a thin PVP layer, diffraction peak intensity increases, indicating the larger pentacene, which is consistent with the former results.

**Figure 3:**
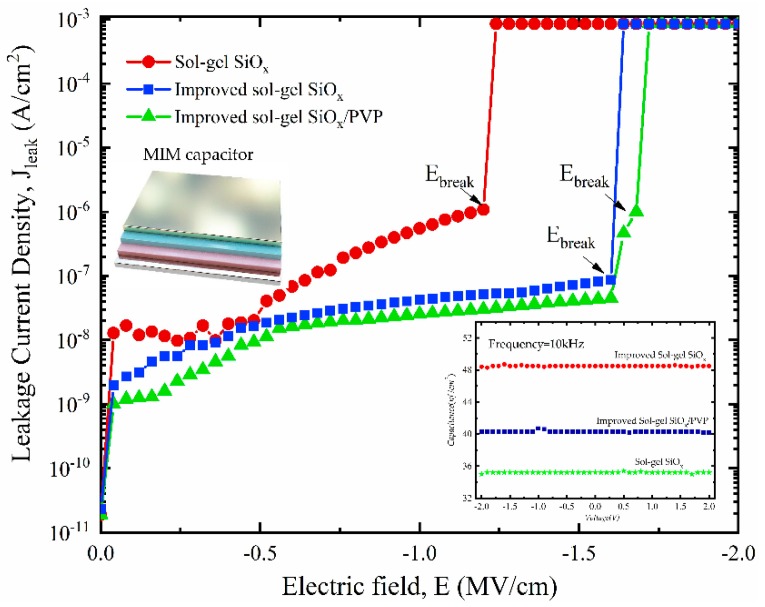
Field-dependent leakage current density for sol–gel SiO*_x_* (~150 nm), improved sol–gel SiO*_x_* (~150 nm), improved sol–gel SiO*_x_*/PVP (~150 + 20 nm). Inset: capacitance of the related MIM capacitor.

In the OTFT devices, the charge density (*Q* = CV) localized at the first few semiconductor monolayers close to the interface is proportional to the dielectric constant [14,15]. The electrical properties of dielectric were determined by measuring a parallel-plate metal–insulator–metal (MIM) capacitor, as plotted in Figure 3. It shows that the leakage current density of improved sol–gel SiO*_x_* capacitor is much lower than the conventional sol–gel process device. The cross-linking PVP further optimized its insulation performance. The capacitor with the hybrid layer also exhibits the highest breakdown electric field (*E_break_*) at ~1.7 MV/cm. It is because, as depicted in Figure 2c,d, the thickness of sol–gel SiO*_x_* is much lower around the holes, and hot spots of the applied electric field are created, resulting in the increase of the leakage current density and the reduced breakdown field strength. The improved film has fewer pores, even before being covered and filled with polymer, which explains the enhanced dielectric property.

**Figure 4:**
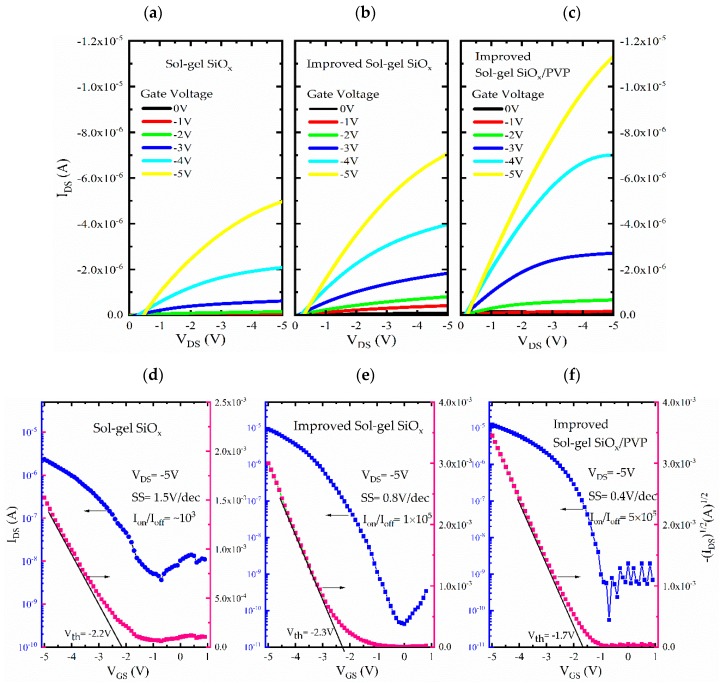
The output and transfer curves of the OTFTs with (**a**,**d**) sol–gel SiO*_x_*, (**b**,**e**) improved sol–gel SiO*_x_*, (**c**,**f**) improved sol–gel SiO*_x_* /PVP dielectric. (**a**), (**b**), (**c**) are in the same scale.

The electrical characteristics of the OTFTs are shown in Figure 4. It can be observed that the electrical properties, such as threshold voltage (*V_th_*), on-off ratio (*I_on_*/*I_off_*) and subthreshold slope (*SS*), of the devices based on different dielectrics vary significantly. Since the bigger the capacitance of the gate insulator is, the more the charge carriers are accumulated at the interface between the organic semiconductor and insulator at a constant voltage, the use of sol–gel SiO*_x_* brings high capacitance that result in the low operated voltage. Although the sol–gel SiO*_x_* sample could operate in a relatively low voltage (*V_th_* = −2.2 V), the holes in the film leads to the increase of leakage current (as shown in Figure S1), and its hysteresis is serious (*I_on_*/*I_off_* = ~10^3^, SS = 1.5 V/dec). The leakage current through the gate dielectric dominates the static power dissipation, and the *SS* value presents how much gate voltage is necessary to increase the drain−source current by one order of magnitude, so it determines total power consumption. By improving the process, the drain current of device with improved sol–gel SiO*_x_* dielectric layer reaches a higher value than that of device with sol–gel SiO*_x_*, a higher on/off ratio (~10^5^), and a lower *SS* (0.8 V/dec). It is because of the lower internal porosity of the dielectric film by the improved process, which promotes the charge capacity (from 35.2 to 48.1 nF/cm^2^). 

The subthreshold slope (*SS*) can be extracted by Equation (1) [3]:

(1) $$SS={\left(\frac{d\log I_{D}}{dV_{G}}\right)}^{-1}$$

The inverse *SS* of the sol–gel SiO*_x_*-based OTFT treated by PVP is 0.4 V/decade, that is three times smaller than that of untreated devices, which is attributed to the lower interfacial trap-site densities and high density of induced charge carriers at the interface of insulators by *V_GS_*. The high capacitance of gate insulators induces the highly efficient charge accumulation process at a constant *V_GS_* that is responsible for the lowered *SS* value. Furthermore, the value of the trap density of the PVP surface also plays a role in *SS*, resulting in that the trap sites can be rapidly prefilled to form conducting channels from source to drain contact bringing a low threshold voltage. Therefore, the low threshold voltage can simultaneously appear with the low *SS* in an OTFT. The interfacial trap-site density (*N_trap_*) may be expressed as:

(2) $$N_{trap}=\frac{C_{i}}{q}\left(\frac{qSS\log e}{k_{B}T}-1\right)$$

where *k_B_* is the Blotzmann’s constant, *T* is the temperature, q is the electronic charge, e is the base of the natural logarithm, *SS* is the subthreshold slope. All established *N_trap_* values are shown in Table 1, from which the *N_trap_* for improved sol–gel SiO*_x_*/PVP sample is reduced by 73%. This can easily explain the reduced threshold voltage and *SS* and the improved field-effect mobility, it also figures out the negligible hysteresis characteristic of the improved devices (as depicted in Figure S2).

**Figure 5:**
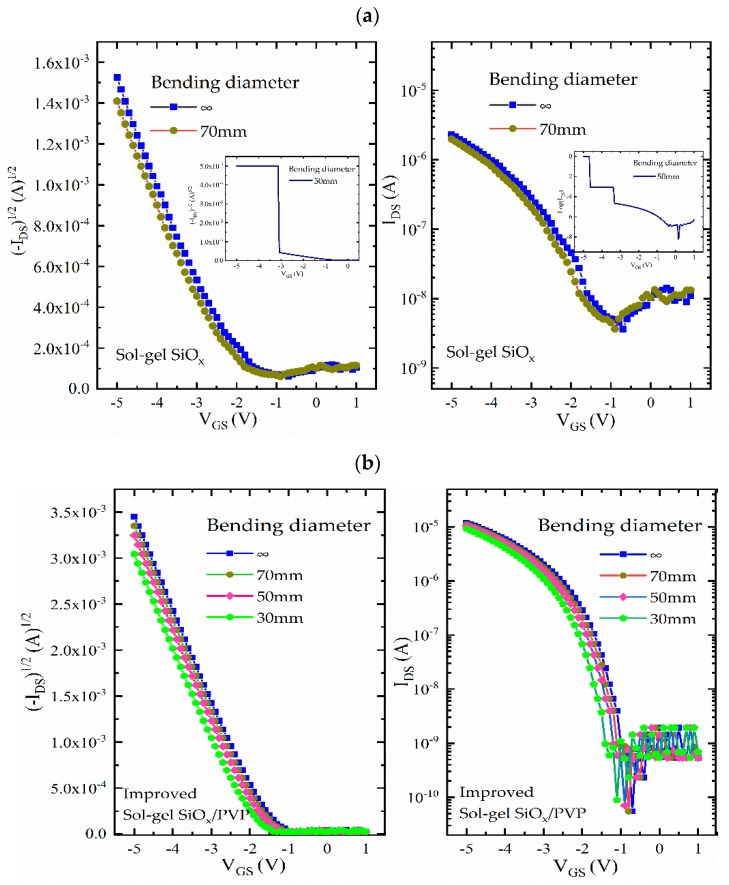
The electrical performance of the flexible OTFTs based on (**a**) sol–gel SiO*_x_*, (**b**) improved sol–gel SiO*_x_*/PVP dielectric with a PI substrate during a stretchable test, applying bending diameter at 70 mm, 50 mm or 30 mm.

To investigate the suitability of the device in flexible applications, the property of transistors in the compressed state was systematically measured. Figure 5 shows the influence of curvature on device electrical performance. For the sol–gel SiO*_x_* sample, the device bent to a diameter of 70 mm and was able to function normally, but when it was tested for greater curvature the bending enlarged the holes and pores in the dielectric, making the device vulnerable to breakdown. Meanwhile, for the improved sol–gel SiO*_x_*/PVP sample, even though there was a negligible shift of operated voltage (increasing from −1.7 V to −1.9 V), no obvious degradation (including its field effect mobility and on/off ratio) was observed at further bending compression, suggesting a good bending stability for the stretchable electronics. 

## 4. Conclusions

In summary, we demonstrated a pentacene based OTFT with the high field effect mobility (1.8 cm^2^/Vs) by adopting a hybrid dielectric consisting of improved solution-processed silica and polyvinyl phenol. The threshold voltage has been significantly reduced, attributed to the high k of sol–gel SiO*_x_*. The *SS* and field-effect mobility were optimized by introducing PVP as the modified layer of sol–gel SiO*_x_* to passivate surface hydroxyl groups, forming a hydrophobic and smoother surface, which led to a better orientation and increased grain size for the pentacene molecules. During the bending test, the results show that the devices based on dielectric fabricated by conventional sol–gel process can be more susceptible to the curvature. The improved device could function well even at the further compression. Current research will be helpful in pushing the practical applications of OTFTs in portable, flexible electronics.

## Fundings

This work was supported by “Program for Outstanding Young Scientific Research Talents in University” funded by Fujian Provincial Department of education, and “Provincial Natural Science Foundation” (2019J01880,2017J01733) funded by Fujian provincial department of science &technology.

## Figures and Tables

**Table 1:** Performance criteria of organic transistors with different dielectrics.

Dielectric Materials	Thickness (nm)	*C_i_* (nF/cm^2^)	*k*	*V_th_* (V)	*I_on_*/*I_off_*	*SS* (V/decade)	*N_trap_* (cm^−2^)
Sol–gel SiO*_x_*	150	35.2	6	−2.2	~10^3^	1.5	5.32 × 10^12^
Improved Sol–gel SiO*_x_*	150	48.5	8.2	−2.3	1 × 10^5^	0.8	3.76 × 10^12^
Improved Sol–gel SiO*_x_*/PVP	170	40.3	7.8	−1.7	5 × 10^5^	0.4	1.44 × 10^12^

## Supplementary Materials

The following are available online at https://www.mdpi.com/2079-4991/10/4/806/s1, Figure S1: The leakage current of the OTFTs based on various dielectric layer by applying the gate voltage from 1 to −5V; Figure S2: The hysteresis characteristic of OTFT device with improved Sol-gel SiOx/PVP dielectric layer; Figure S3: The normalized D/S current of OTFT device with improved sol–gel SiOx/PVP dielectric layer when constant bias stress *V_GS_* = −5V is applied for more than 3 h.
