# Supplementary material for: Facile Synthesis of Solution-Processed Silica and Polyvinyl Phenol Hybrid Dielectric for Flexible Organic Transistors
Source: Nanomaterials (Basel). 2020 Apr 23;10(4):806. doi: 10.3390/nano10040806 (PMC7221767; doi:10.3390/nano10040806)
Supplement: Supplementary file 1 [file nanomaterials-10-00806-s001.pdf]

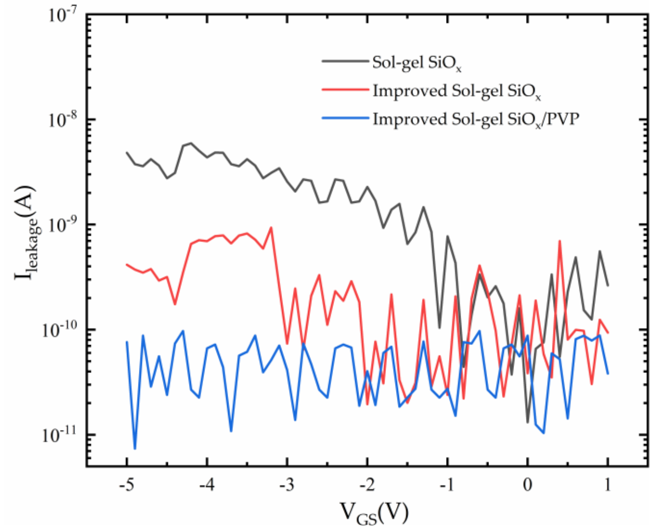

Figure S1. The leakage current of the OTFTs based on various dielectric layer by applying the gate voltage from 1 to −5V.

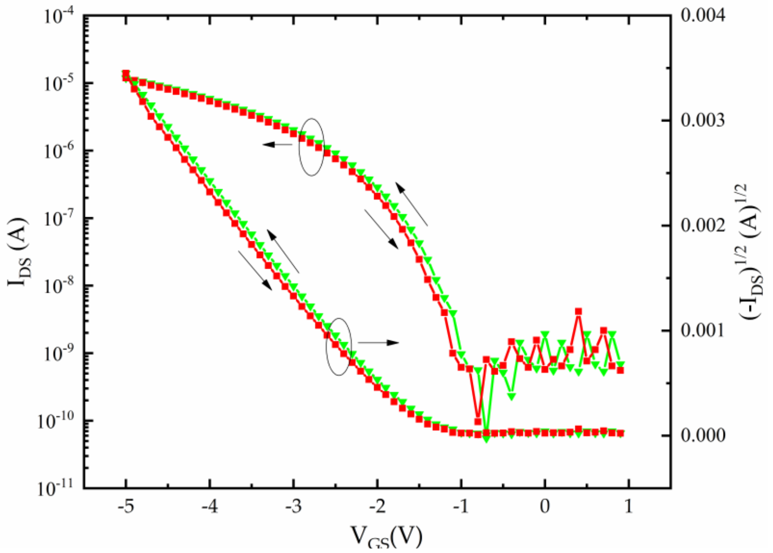

Figure S2. The hysteresis characteristic of OTFT device with improved sol–gel SiO_x/PVP dielectric layer.

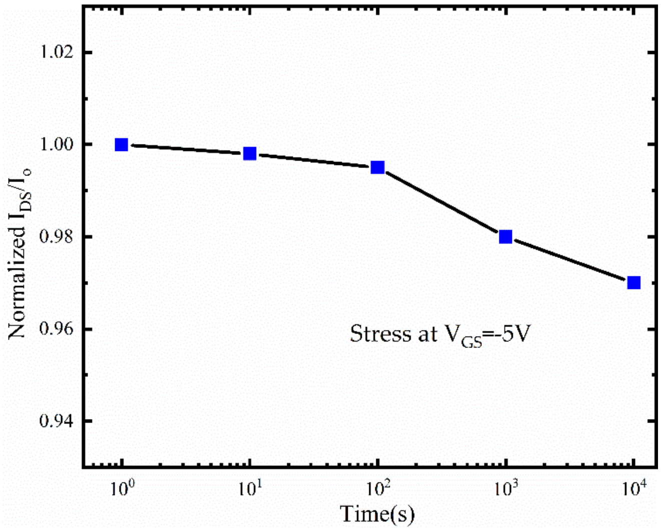

Figure S3. The normalized D/S current of OTFT device with improved Sol-gel SiO_x/PVP dielectric layer when constant bias stress $V_{GS} = −5V$ is applied for more than 3 h.
